# Primary Dermal Melanoma: A Rare Subtype of Melanoma and Clinical Mimicker of Basal Cell Carcinoma

**DOI:** 10.7759/cureus.65538

**Published:** 2024-07-27

**Authors:** Kelly E Owens, Ysaac Zegeye, Rayan Saade, Michelle B Pavlis

**Affiliations:** 1 Department of Dermatology, Duke University School of Medicine, Durham, USA; 2 Department of Pathology, Duke University School of Medicine, Durham, USA; 3 Department of Dermatology, Durham Veterans Affairs Medical Center, Durham, USA

**Keywords:** melanoma surgery, ­melanoma and nevi, ­skin cancer, metastatic skin cancer, skin cancer detection, solitary dermal melanoma, diagnostic uncertainty, metastatic melanoma, melanoma, primary dermal melanoma

## Abstract

Primary dermal melanoma (PDM) is a rare variant of melanoma. We present the case of a 76-year-old female diagnosed with PDM following initial suspicion of basal cell carcinoma, prompting extensive workup to exclude metastasis. This case demonstrates the diagnostic challenges and need for rigorous evaluation in suspected PDM cases. Current literature lacks definitive diagnostic markers for PDM, and we highlight the ongoing need for research and collaborative efforts between dermatology and oncology.

## Introduction

Primary dermal melanoma (PDM) is a rare and distinct subtype of melanoma that is characterized by its localization to the dermis or subcutaneous tissue without any epidermal involvement. This subtype represents a unique clinical entity that can be challenging to diagnose due to its atypical presentation. Unlike other forms of melanoma that typically arise in the epidermis, PDM often presents without the typical pigmented lesion, making it difficult to identify through visual examination alone. This distinction is critical as PDMs frequently present clinically as amelanotic, lacking the pigmentation usually associated with melanomas, which can lead to misdiagnosis. They can mimic various other benign and malignant conditions, such as subcutaneous nodules, cysts, or basal cell carcinomas (BCC), making histopathological differentiation essential. Understanding the unique characteristics of PDM is important for ensuring accurate diagnosis and appropriate management. Recent advancements in immunohistochemistry and genetic profiling, such as the use of the melanoma hotspot next-generation sequencing panel, have improved the diagnostic accuracy for these challenging cases [1,2].

## Case presentation

**Figure 1:**
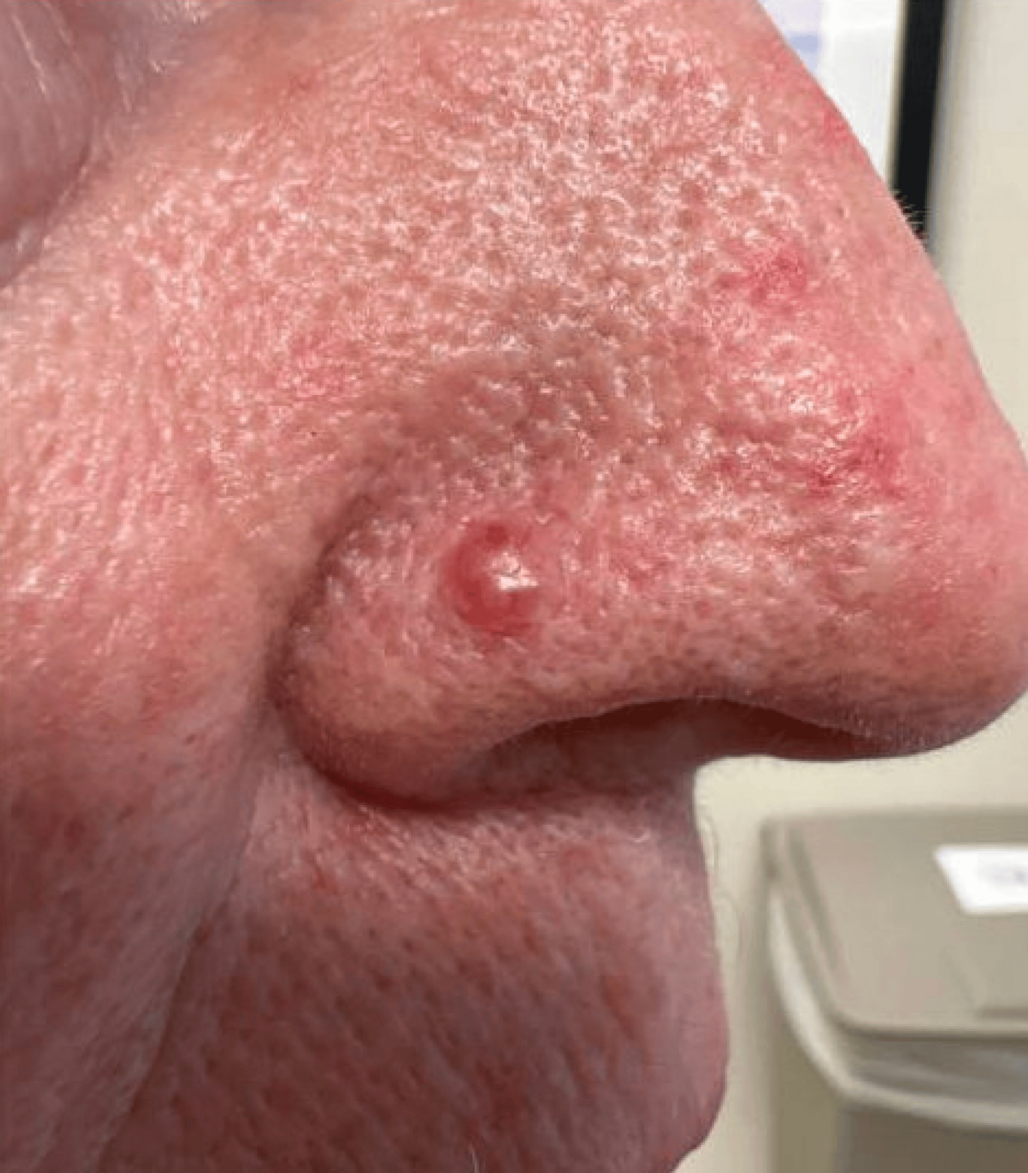
A 6-mm pearly, erythematous papule on the right nasal ala with arborizing vessels

A 76-year-old female patient with a history of numerous BCCs, squamous cell carcinomas (SCCs), and actinic keratoses presented with a newly enlarging papule on the right nasal ala. The patient noticed it a few weeks prior and had no associated symptoms other than an increase in size. She underwent a full body skin examination (FBSE) just one month earlier, with no suspicious lesions noted. On examination, a 6-mm pearly, erythematous papule with arborizing vessels was noted on the right nasal ala (Figure 1), raising high clinical suspicion for BCC.

**Figure 2:**
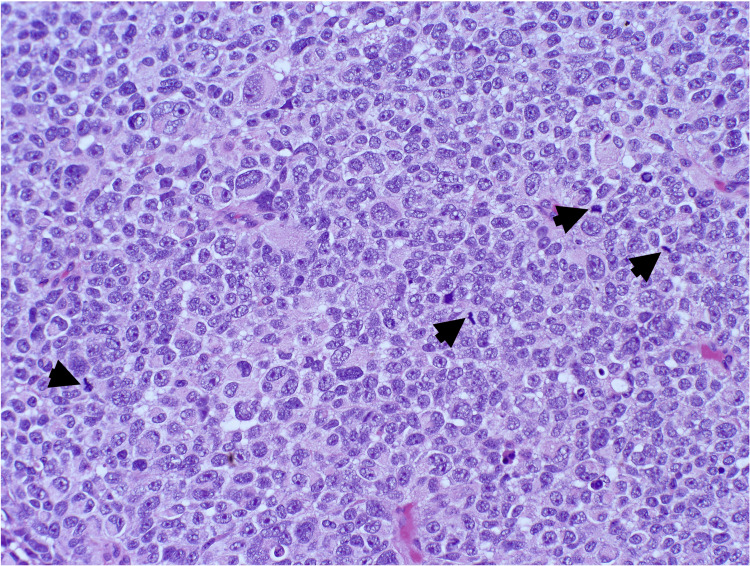
Melanocytes showing severe cytologic atypia represented by large pleomorphic nuclei with irregular contours, prominent nucleoli, and numerous mitotic figures (black arrowheads) (H&E, 200×) H&E: hematoxylin and eosin

**Figure 3:**
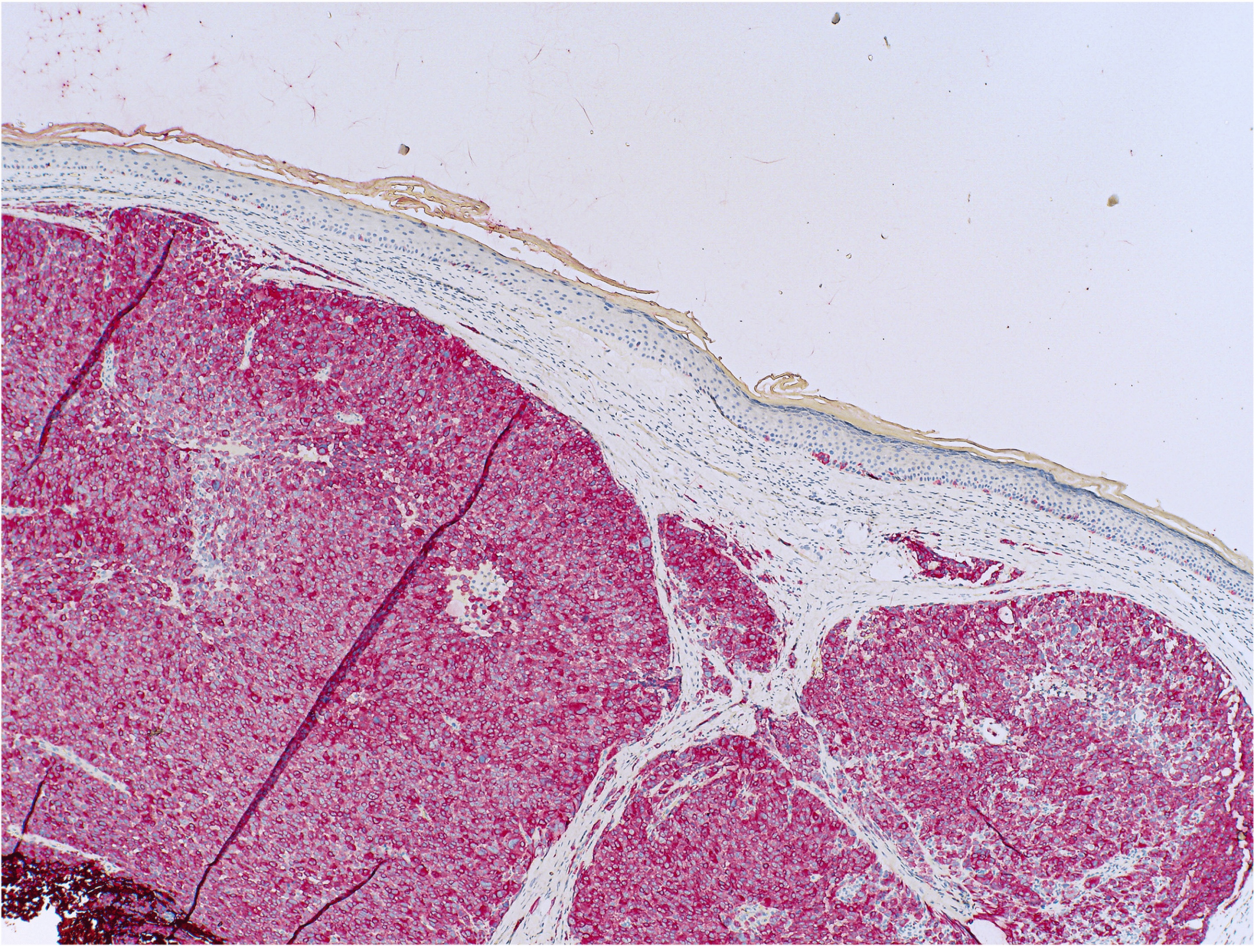
The tumor cells that are diffusely positive for the MART-1 IHC study (40×) MART-1: melanoma antigen recognized by T-cells 1; IHC: immunohistochemical

**Figure 4:**
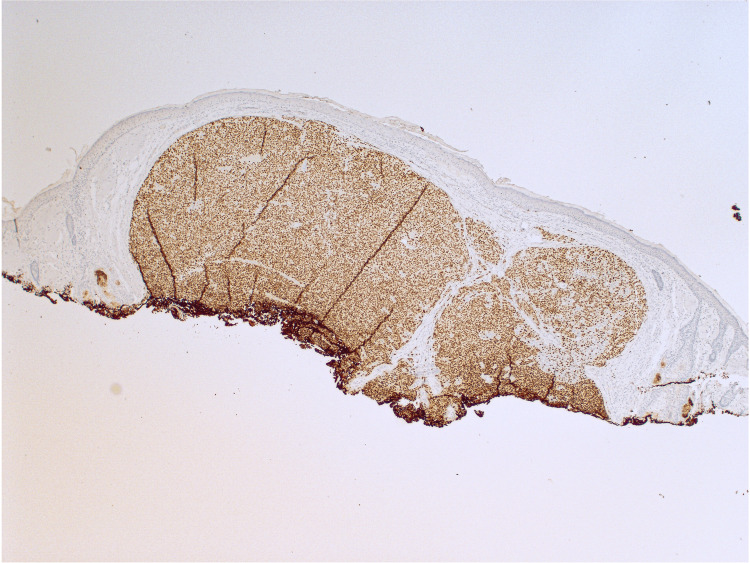
PRAME is positive in tumor cells and shows >99% nuclear expression (IHC, 100×). PRAME: preferentially expressed antigen in melanoma; IHC: immunohistochemical

Shave biopsy demonstrated a dermally based proliferation of large nodules with pleomorphic and hyperchromatic nuclei, lacking overlying atypical junctional melanocytic proliferation (Figure 2). Immunohistochemistry markers were positive for melanoma antigen recognized by T-cells 1, SRY-box transcription factor 10, and preferentially expressed antigen in melanoma and negative for Ber-EP4 (epithelial cell adhesion molecule), p40 (a marker for SCC), insulinoma-associated protein 1, and adipophilin (a lipid droplet-associated protein) (Figures 3, 4). Melanoma prognostic factors included a Breslow depth of 2 mm, a mitotic rate of 25 figures per mm^2^, and no regression or ulceration. Pathology favored a differential diagnosis of either a metastatic melanoma deposit or a PDM, prompting referral to medical oncology. Further evaluation revealed no signs of metastasis, including a negative melanoma hotspot NGS panel, sentinel lymph node biopsy (SLNB), and positron emission tomography-computed tomography (PET-CT) scan.

One month after the initial presentation, the patient underwent Mohs micrographic surgery, resulting in the excision of the entire alar subunit following the removal of a debulking specimen. Histological examination showed no residual melanoma at the lateral and deep margins. Reconstruction was performed with a pedicled nasolabial interpolation flap and cartilage graft from the right antihelix. The patient is scheduled for CT scans at six-month intervals for metastatic surveillance and FBSE every four months. After 18 months, there has been no local recurrence or metastasis.

## Discussion

PDM was first formally recognized by Bowen et al. and represents 1% of all melanoma cases [2,3]. Soon after, Swetter et al. proposed initial PDM criteria in an effort to distinguish PDM from a metastatic melanoma deposit [2]. This criteria is widely cited and includes the following components: (1) a dermal-based melanocytic neoplasm, typically with nodular or multinodular architecture, (2) well-accepted features of malignancy, including cytologic atypia, numerous mitoses, and areas of necrosis, (3) no evidence of an intraepidermal (in situ) component even with multiple sections, (4) absence of ulceration, (5) positive findings for S100, (6) absence of continuity with large peripheral nerves, (7) absence of a preexisting melanocytic nevus, and (8) absence of regression [2].

Clinically, PDM is difficult to diagnose due to its varied presentation, which may mimic benign lesions and nonmelanoma skin cancers such as cysts, inflammatory papules, BCC, or Merkel cell carcinoma, rather than melanocytic neoplasms. In fact, initial clinical assessments frequently overlook melanoma, as evidenced by this case report and other studies where melanocytic neoplasms were not considered in the differential diagnosis of PDM cases [2,4]. This clinical uncertainty can lead to unexpected diagnoses, transforming routine dermatologic management into complex oncologic pathways. Referral to surgical oncology and medical oncology for staging evaluations, including SLNB and PET-CT imaging, becomes relevant to exclude metastatic disease and guide therapeutic strategies. One study evaluating the utility of SLNB in PDM indicated its superiority over simple excision alone, demonstrating greater utility, with determinants being disease-free survival, overall survival, progression, and cost-effectiveness [5]. However, more studies are needed alongside the assessment of independent risk factors.

Despite attempts to find immunohistochemical staining differences that distinguish PDM from metastatic melanoma, definitive markers do not exist. Current research highlights markers such as S100, human melanoma black 45, and Ki-67 positivity in PDM, which are indistinguishable from other melanoma types [2,6]. These challenges display the difficulty in differentiating PDM histologically and clinically from metastatic melanoma, inciting discussion over its management as a distinct entity.

The pathophysiology of PDM is not well understood. Still, some authors suspect PDM may represent de novo melanoma from ectopic melanocytes, melanoma with a regressed epidermal component, or a metastatic melanoma deposit from a regressed primary. Regardless, improved survival outcomes compared to metastatic melanoma suggest unique biological behaviors that may warrant individualized therapeutic approaches [7].

In clinical practice, dermatologists face dual challenges: discerning whether a lesion represents melanoma and then whether it constitutes PDM or metastatic disease. This necessitates clear communication with patients regarding diagnostic uncertainties, treatment options, and multidisciplinary collaboration.

## Conclusions

PDM poses significant diagnostic and therapeutic challenges in dermatology. Due to its rarity and lack of identifying factors, PDM is often a diagnosis of exclusion, requiring physicians to rule out regional, nodal, and distant metastasis first. Here, we presented a case that demonstrates the complexities in obtaining a PDM diagnosis and the unexpected evaluations and surveillance that ensues. Continued research efforts are essential to better understand PDM and refine its clinical management.
